# Large bowel perforation due to cytomegalovirus colitis in a post-cardiac surgical patient on chronic hemodialysis

**DOI:** 10.1186/s44215-023-00078-7

**Published:** 2023-08-01

**Authors:** Naoto Fukunaga, Tatsuto Wakami, Akio Shimoji, Toshi Maeda, Otohime Mori, Kosuke Yoshizawa, Tatsuji Okada, Nobushige Tamura

**Affiliations:** Department of Cardiovascular Surgery, Hyogo Prefectural Amagasaki General Medical Center, 2-17-77, Higashinaniwa-Cho, Amagasaki, Hyogo 660-8550 Japan

**Keywords:** Cytomegalovirus colitis, Colon perforation, Cardiac surgery

## Abstract

**Background:**

Cytomegalovirus (CMV) colitis with colonic perforation is an exceedingly rare but life-threatening condition. The most comorbid diagnosis in patients with perforated CMV colitis is human immunodeficiency virus in the setting of advanced immunosuppression associated with CD4 < 50 cells/μL.

**Case presentation:**

A 75-year-old female with a ≥ 30-year history of hemodialysis presented with progressive dyspnea on exertion. Transthoracic echocardiography showed moderate mitral stenosis and severe aortic stenosis. Although she was a high-risk candidate with a risk for mortality of 17.17% and morbidity and mortality both of 35.63% in The Society of Thoracic Surgeons risk calculator, we performed mitral and aortic valve replacement with both biological valves. Postoperative course was complicated with a high dose of inotropic support, cardiac tamponade requiring open drainage, and the need for a tracheostomy. Abdominal distension was observed, and enhanced computed tomography demonstrated free air and a suspected perforated sigmoid colon. Emergency laparotomy revealed a 20-cm longitudinal perforation in the sigmoid colon. A left hemicolectomy with stoma was performed. Immunostaining of a sample of her colon showed cytomegalovirus-positive cells.

**Conclusions:**

Cardiac surgeons should consider cytomegalovirus colitis as a differential diagnosis during the course of cardiac surgery even in immunocompetent patients.

## Background

Cytomegalovirus (CMV) colitis with colonic perforation is an exceedingly rare but life-threatening condition. The most comorbid diagnosis in patients with perforated CMV colitis is human immunodeficiency virus (HIV) in the setting of advanced immunosuppression associated with CD4 < 50 cells/μL [1, 2]. We herein report a case of sigmoid colon perforation associated with CMV colitis in a post-cardiac surgical patient. Although this is an unusual situation, it can nevertheless occur in an immunocompetent cohort.

## Case presentation

A 75-year female with a ≥ 30-year history of hemodialysis for chronic glomerular nephritis presented with progressive dyspnea on exertion. She had been followed for progressive mitral and aortic stenosis at our cardiology unit. Hyperlipidemia was evident, and her surgical history included permanent pacemaker implantation for atrioventricular block 1 year previously. Her medication did not include immunosuppressive agents.

Recently, she showed poor tolerance of hemodialysis because of low blood pressure without inotropic support. Transthoracic echocardiography showed moderate mitral stenosis with a mean pressure gradient of 7.7 mmHg and valve area of 1.07 cm^2^. Severe aortic stenosis with a mean pressure gradient of 35 mmHg, peak velocity of 4.4 m/sec, valve area of 0.90 cm^2^, and a preserved left ventricular function were also observed. Mitral annular calcification was evident in the posterior wall of the left atrium and the posterior leaflet. Enhanced computed tomography (CT) revealed minor calcification of coronary arteries, calcification of the entire aorta, and mitral annular calcification in both leaflets. The Society of Thoracic Surgeons (STS) short-term risk calculator indicated a risk of mortality of 17.17% and morbidity and mortality both of 35.63%.

Despite high surgical risk, the patient desired cardiac operation. We performed chordal sparing mitral valve replacement, debridement of mitral annular calcification, and aortic valve replacement with both biological valves. Intraoperative transesophageal echocardiography demonstrated the well-functioning biological valves. On weaning from cardiopulmonary bypass, she required high dose of inotropic support and was critically ill in the intensive care unit. Duration of aortic cross clamp and cardiopulmonary bypass was 265 min and 347 min, respectively. Postoperative transthoracic echocardiography showed well-functioning valves without paravalvular leakage in both positions. Left ventricular ejection fraction was 61% with normal left ventricle. Her postoperative course was complicated by prolonged inotropic support, ventilation requiring tracheostomy, and cardiac tamponade requiring open drainage. During the stay of intensive care unit, several large-amount transfusions were administered to maintain hemodynamics. Inflammatory reaction on blood work had been slightly elevated, but there was no clear evidence of infection. We submitted blood cultures, which were all negative. The patient was also negative for Clostridium difficile as she had loose stool and was given antibiotics for several days. On postoperative day 29, abdominal distension was observed suddenly. Patient required high volume resuscitation for hypotension and sepsis. We suspected non-occlusive mesenteric ischemia (NOMI) in the context of hemodialysis. Enhanced CT demonstrated the patency of superior mesenteric artery (SMA) without evidence of narrowing [3], free air and a suspected perforated sigmoid colon. There were no findings to suspect NOMI such as hepatic portal venous gas, pneumatosis intestinalis of bowel wall, and gas in inferior vena cava and superior mesenteric vein [3]. A diagnosis of bowel perforation was made.

**Fig. 1:**
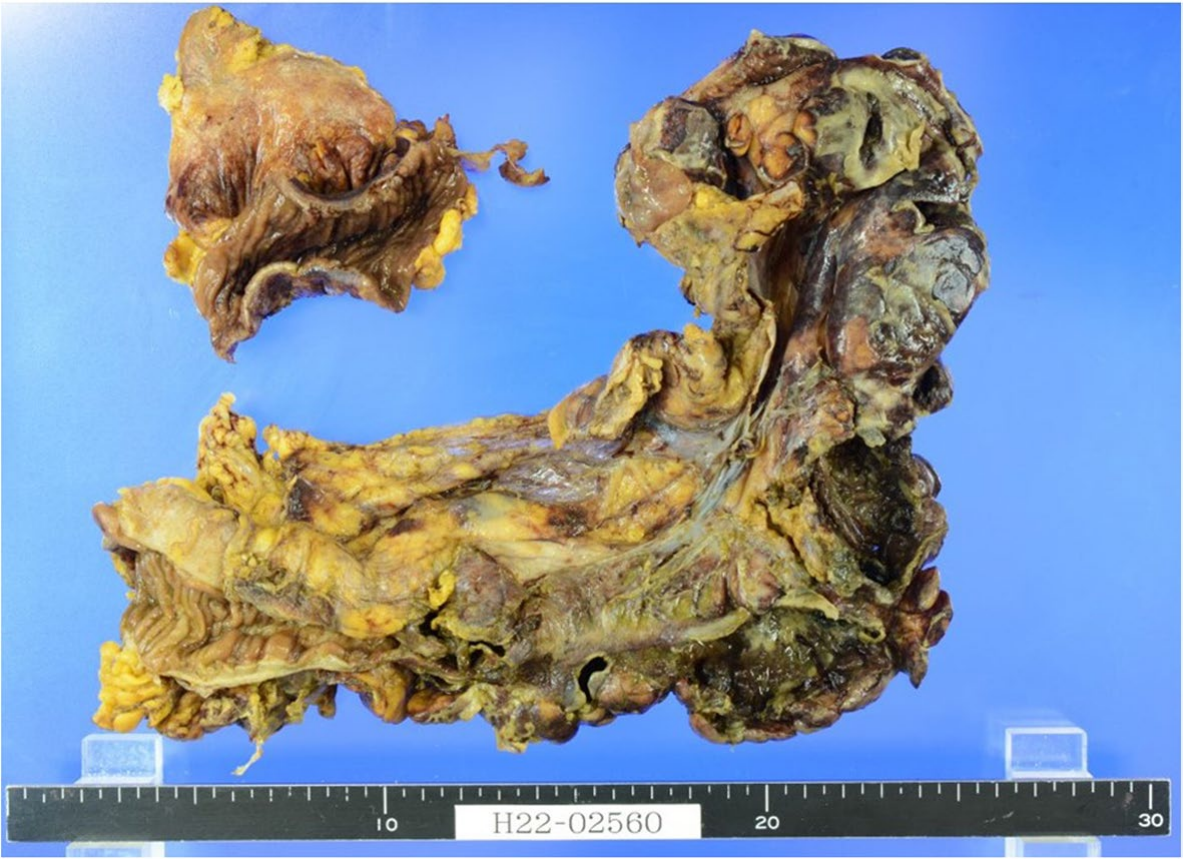
A submitted specimen of the sigmoid colon is already open. It shows the large area of inflammation

**Fig. 2:**
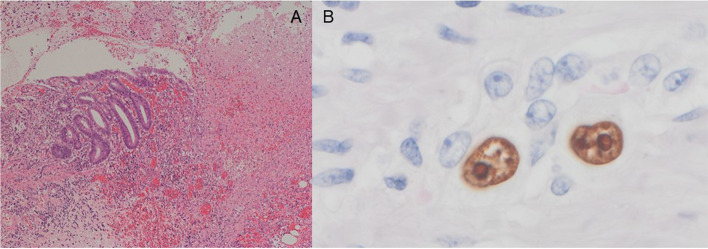
**A** A specimen shows severe inflammation with neutrophilic infiltration in the sigmoid colon (H/E stain, × 100). **B** Immunostaining of perforated colon shows cytomegalovirus-positive cells

Emergency laparotomy by general surgeons revealed a 20-cm longitudinal perforation in the sigmoid colon with large quantities of watery stool inside the abdomen (Fig. 1). A left hemicolectomy with stoma was performed, and the patient was returned to the intensive care unit on high dose inotropic support. Pathological finding of the submitted specimen showed severe and extensive inflammation with neutrophilic infiltration in the sigmoid colon. There was no evidence of ischemia (Fig. 2A). Additional immunostaining of a sample of her colon showed CMV-positive cells (Fig. 2B).

Her postoperative course following laparotomy was complicated with a high dose of inotropes and refractory sepsis. Unfortunately, she was not responsive to our treatment including polymyxin B hemoperfusion, and her family decided to withdraw care after a long discussion. She died of sepsis secondary to the perforated sigmoid colon 10 days after her laparotomy.

## Discussion and conclusions

Most instances of CMV infection occur in immunocompromised patients who have advanced HIV infection with severe immunosuppression and malignant tumors, and who have undergone organ transplants and chemotherapy [2, 4]. Although our patient was a high-risk candidate based on her preoperative background with a STS mortality score of over 17%, she took no immunosuppressive agents and did not have a history of immunosuppressive disease.

NOMI is a post-cardiac surgical complication with a high mortality rate that develops from a vasospasm related to low perfusion of the SMA during cardiopulmonary bypass or intra/postoperative high dose of inotropes, as well as other risk factors [5]. Quantitative analysis of SMA calcification in hemodialysis patients could be a predictive marker for acute mesenteric ischemia, including NOMI. NOMI occurs more frequently in hemodialysis patients [6]. Considering the calcification of the entire aorta and long-term hemodialysis of our patient, we strongly suspected NOMI when she developed abdominal distension, but there was no evidence of it. Although CT findings on NOMI were poorly understood, there were no findings to suspect NOMI such as hepatic portal venous gas, pneumatosis intestinalis of bowel wall, and gas in inferior vena cava and superior mesenteric vein. To confirm NOMI, the selective angiography of SMA is required [3]. Thus, we denied NOMI in our case.

Instead, we encountered sigmoid perforation secondary to CMV colitis during cardiac surgery. CMV colitis remains a diagnostic challenge [2], and is not usually suspected in the absence of advanced immunosuppression. To identify CMV colitis, colonoscopy is preferred. CMV colitis may appear as colitis alone, colitis with ulceration or aggregates of discrete ulcers surrounded by unremarkable mucosa by endoscopy. For a diagnosis of CMV colitis, identification of typical inclusion bodies in the tissue samples is necessary [7]. We denied the common NOMI with the abovementioned reasons, and identified the inclusion bodies in the submitted samples. Thus, a diagnosis of CMV colitis was made.

Fourteen patients with end-stage renal disease, nine of which were on dialysis, may have the higher risk of CMV infection due to frequent blood transfusion and contaminated dialysis equipment [8]. Indeed, red blood cell transfusion within one month of the diagnosis of CMV colitis was found to be an independent risk factor for the development of CMV colitis in immunocompetent patients [9]. The study also found more patients on hemodialysis in CMV colitis group compared with the control group (*P* = 0.003).

Siciliano et al. studied 14 previously immunocompetent patients in intensive care unit patients with CMV colitis and end-stage renal disease, nine of whom were on dialysis. A transient depression in immunity, predisposing individuals to viral reactivations, is a possible mechanism of CMV infection in a critically ill setting [10]. The gastrointestinal tract was the most common site of involvement in these patients [2], and the clinical manifestation was lower gastrointestinal bleeding [8, 10]. Irrespective of specific treatment with ganciclovir, in-hospital mortality rate was high. Lopez-Rao et al. reviewed non-immunosuppressed heart surgery patients excluding dialysis patients to investigate the prevalence of CMV infection in the intensive care unit. Surprisingly, CMV reactivation was observed in 16.5% patients undergoing heart surgery at a median of 17 days following admission to the intensive care unit. In addition, CMV reactivation was independently associated with continued hospitalization or death by day 30 [11]. This study was practical as it casts an important point that CMV reactivation frequently occurs in heart surgery patients, and we all have to recognize the possibility of CMV reactivation in patients who stay in the intensive care unit longer.

Our patient was critically ill in the postoperative period. She had received high-dose of inotropes to maintain hemodynamic stability, had been intubated for several weeks, was on hemodialysis, and had received several large-volume transfusions. Based on the abovementioned findings, she therefore had a number of risk factors for developing a CMV infection despite its rarity. To our knowledge, this is the first report of CMV colitis and related perforation after heart valve replacement in a patient on dialysis.

In conclusion, CMV colitis should be suspected following clinical signs such as gastrointestinal bleeding or acute abdominal symptoms in a critically ill or post-surgical setting, and as a differential diagnosis for NOMI. In addition, we have to take into consideration the frequent incidence of CMV reactivation after cardiac surgery.

## Data Availability

Data sharing is not applicable to this article as no datasets were generated or analyzed during the current study.

## Abbreviations

CMV: Cytomegalovirus
CT: Computed tomography
HIV: Human immunodeficiency virus
NOMI: Non-occlusive mesenteric ischemia
SMA: Superior mesenteric artery
STS: The Society of Thoracic Surgeons
